# 

**Published:** 1890-03

**Authors:** 


### CONTENTS.

ORIGINAL COMMUNICATIONS—

Coccygodynia. By Hunter P. Cooper, M. D., Atlanta, Ga........... 1

Treatment of Chancroid by the Potassate of Sozoiodol. By Charles Szadek, M. D., Kieff, Russia........................................ 10

Potassii Nitras in the Treatment of Chill and Fever—(Clinical Notes, page 54.) By J. D. Hunter, M. D., New Orleans, La.. 12

Obstetrics and Gynecology. By E. S. McKee, M. D., Cincinnati ................................................................................ 14

A Case of Adherent Placenta. By Isham Harrison, M. D., Deerbrook, Miss ........................................................... 18

Conservative Treatment of Inflammatory Diseases of the Uterine Appendages and Sequelæ by Electricity. By Augustin H. Goelet, M. D., New York... ......................... 20

SOCIETY REPORTS—

Philadelphia Obstetrical Society........................ .............. 29

Atlanta Society of Medicine............................................ 43

CORRESPONDENCE—

Our New York Letter..................... ...................................... 48

Letter from A. F. Barnard, M. D........................................ 54

CLINIC REPORTS—

Report of Case of Entozoa in the Kidney. By W. J. Greene, M. D., Fort Valley, Ga....................................................... 55

BOOK REVIEWS.......................................................... 57

EDITORIAL—

The Treatment of Pyosalpinx........................................... 59

Items.......................................................................... 61

On two cases of Poisoning by Anilides (Exalgine and Antifebrin) ................. ............................................................ 62

